# Correspondence of aCGH and long-read genome assembly for detection of copy number differences: A proof-of-concept with cichlid genomes

**DOI:** 10.1371/journal.pone.0258193

**Published:** 2021-10-07

**Authors:** Gabriel A. Preising, Joshua J. Faber-Hammond, Suzy C. P. Renn

**Affiliations:** Department of Biology, Reed College, Portland, OR, United States of America; Laboratoire de Biologie du Développement de Villefranche-sur-Mer, FRANCE

## Abstract

Copy number variation is an important source of genetic variation, yet data are often lacking due to technical limitations for detection given the current genome assemblies. Our goal is to demonstrate the extent to which an array-based platform (aCGH) can identify genomic loci that are collapsed in genome assemblies that were built with short-read technology. Taking advantage of two cichlid species for which genome assemblies based on Illumina and PacBio are available, we show that inter-species aCGH log_2_ hybridization ratios correlate more strongly with inferred copy number differences based on PacBio-built genome assemblies than based on Illumina-built genome assemblies. With regard to inter-species copy number differences of specific genes identified by each platform, the set identified by aCGH intersects to a greater extent with the set identified by PacBio than with the set identified by Illumina. Gene function, according to Gene Ontology analysis, did not substantially differ among platforms, and platforms converged on functions associated with adaptive phenotypes. The results of the current study further demonstrate that aCGH is an effective platform for identifying copy number variable sequences, particularly those collapsed in short read genome assemblies.

## Introduction

Cichlid fishes have long been a model biological system to study evolutionary processes given their phenotypic diversity, recent radiations, and high propensity for speciation [1, 2]. The initial sequencing of 5 cichlid genomes has paved the way for investigations on the genomic correlates of adaptive radiations [3, 4], and the results highlighted the importance of both sequence and structural variation between taxa [4, 5]. Given these genomes were assembled using short-read sequencing technology, which is prone to collapsing repetitive genomic regions with high shared sequence identity [6], this made it difficult to accurately quantify copy number differences between species as well as variation within species. Limitations of short-read read-depth approaches for quantification of copy number differences [7] necessitated the complementary approach of array-based comparative genomic hybridization (aCGH) [8].

Long-read genome assemblies use single-molecule sequencing that can recover the otherwise collapsed regions in short-read assemblies [9]. The releases of PacBio assemblies for *Metriaclima zebra* and *Oreochromis niloticus* present an opportunity to quantify inter-species copy number differences across multiple platforms [10–12]. aCGH has been shown to capture recent gene duplications that can be highly repetitive (*e*.*g*. human: [13]; *Drosophila*: [14]; yeast: [15]). Faber-Hammond et al. (2019) used aCGH to quantify intra-species copy number variation (CNV) as well as inter-species copy number differences between 53 species across 12 tribes of African cichlids [8]. This study showed an average of 50–100 CNVs per individual with a high degree of intra-species variation such that only ∼10% of detected copy number variable sites appeared fixed for a given species. The authors also found that 4.5 times more CNV-related aCGH probe sequences aligned to more *O*. *niloticus* PacBio assembly loci than to Illumina assembly loci whereas random probe sequences had a significantly lower (1.75:1) ratio.

Here, we used two cichlid species to perform an investigation into the concordance between copy number detection approaches, which include using aCGH data and available Illumina and PacBio assemblies. We found a strong correlation between inter-species aCGH log-fold ratios and PacBio sequence copy numbers, and we bioinformatically validate the gene pathways that were found to be copy number variable in the prior study. Although each copy number detection platform has its own strengths and weaknesses, this work presents a further proof-of-concept for the techniques applied in aCGH studies by demonstrating that aCGH-detected inter-species copy number differences are supported by sequencing technology as genome assemblies improve [8, 16–18].

## Methods

### Inter-species aCGH log_2_ hybridization ratio

aCGH involves competitively hybridizing fluorescently-labeled DNA from different samples to probes on a microarray. By measuring the relative fluorescence from each sample, one can ascertain the relative copy numbers of the DNA segment represented by a probe. To identify and quantify copy number differences between species we performed inter-species competitive aCGH using the 12-plex custom Cichlid array (GEO accession GPL25405). The array was constructed such that each gene is represented by 3 probes, with intergenic probes roughly every 6 kb. Notably, the array has an *O*. *niloticus* bias due to the poorly assembled genomic regions being absent in other cichlid species and probe sequences being designed primarily based on the high-quality *O*. *niloticus* genome [8]. We used three different *M*. *zebra* samples (Mz #1, #2, #3 wild caught; provided by Tom Kocher) derived from different fish than were used to build the genome assembly and genomic DNA from the University of Stirling *O*. *niloticus* (On) clonal line [19] previously used for genome assembly (see below). Genomic DNA isolated from ethanol-preserved fin or muscle tissue was labeled with NimbleGen dual-color kits and competitively hybridized in equal amounts in a reference design of Cy3 and Cy5 to the array for 64 hours at 42 °C (NimbleGen Hybridization Station 4) prior to rinsing and scanning (GenPix 4000 Scanner 5 μm/pixel resolution). Dual-color signal-intensity matrices (GEO: GSE141976) were analyzed in RStudio v3.1.3. Four different arrays were used, thus, the data represent 3 biological replicates and one technical replicate (Mz #1 vs On, Mz #2 vs On, Mz #3.1 vs On, and Mz#3.2 vs On). Data were processed through the DEVA v.2.1 (NimbleGen) pipeline with q-spline normalization and exported for GC-Loess normalization in R-Studio v3.1.3 using Ringo and limma packages [20, 21]. We calculated weighted median log_2_ hybridization ratios for each probe by averaging among two replicate arrays and then finding the median hybridization ratio of that and two other unique competitive hybridizations. We used this inter-species aCGH log_2_ hybridization ratio for further analyses and set a +/-0.8 threshold for calling inter-species copy number difference. This threshold was previously validated to optimize the strength of correlation and decrease the number of discordant probes between aCGH and independent read-depth-based data [8].

### Inter-species genome assembly log_2_ hit ratio

To obtain a sequence-based assessment of inter-species copy number differences that could be compared to aCGH results, we identified the number of hits in each genome assembly for the probe sequences on the same 12-plex custom cichlid array described above (GEO accession GPL25405). Probe sequences were aligned to *O*. *niloticus* and *M*. *zebra* and short-read Illumina (RefSeq accession GCA_000188235.2, GCF_000238955.1) and long-read PacBio (GCF_001858045.2, GCF_000238955.4) genome assemblies using BLASTn (e-value = 10) and filtered conservatively for perfect alignments (100% identical full probe length) appearing at least once in each assembly. To establish a metric comparable to the inter-species log_2_ aCGH hybridization ratio, we calculated inter-species genome assembly log_2_ hit ratios for each probe representing the number of perfect alignments for probes in *O*. *niloticus* assembly relative to *M*. *zebra* for both PacBio and Illumina assemblies. While robust copy number detection methods based on read-depth are available (e.g. mrCaNaVAR; CNVnator [22]), we used exact match sequence copy numbers for several reasons. First, the sequence match approach allowed direct one-to-one comparison to the corresponding loci for specific probes used in aCGH as opposed to comparing to a mean depth across a larger genomic interval. Second, sequence matches allowed us to avoid complications with varying overall read depth between species and this approach avoided biases of read depth algorithms optimized for specific technologies (e.g. library preparation and asynchronous amplification [22, 23]). A 100% BLASTn alignment threshold was selected because it allowed for nucleotide composition analysis of probes detected by each sequencing platform (see below). Decreasing BLASTn alignment thresholds below 100% did not appreciably improve relative copy number concordance across platforms (S1 Fig).

### Inter-platform hit bias

To quantify the number of loci that show differential copy number detection across the NGS platforms and assemblies, we identified the subsets of probes that have positive or negative inter-platform log_2_ hit ratios using the number of perfect hits in the PacBio assembly compared to the number of perfect hits in the Illumina assembly within each of the two species.

### Statistical analyses

We performed all statistical analysis comparing probe inter-species log_2_ ratios from each of the three technologies in R-studio v1.1.383 [24]. Pearson’s R was calculated to test significance and strength of correlations in pairwise dataset comparisons using the Stats R package v3.5.1. Pairwise correlation analyses were run for all probes that represent putative multi-copy sequences by having two or more hits in at least one assembly and sets of probes detected as copy number gains in either species by each pair of platforms. Probe sequence nucleotide composition (nucleotide frequencies, dinucleotide frequencies, and overall G/C and A/T) was assessed using Mesquite v3.6 [25] and pairwise permutation MANOVAs were performed on distance matrices built from nucleotide statistics for sets of probes identified as gains by each of the three technologies (RVAideMemoire v0.9–73 [26]). Post-hoc ANOVAs were run between pairs of platform-exclusive probe sets using all possible nucleotide/dinucleotide characteristics to identify those that account for pairwise platform biases. We also performed principal component analysis (PCA) to examine the relative contributions of nucleotide frequencies across the dataset (Stats R package). aCGH log_2_ hybridization ratios associated with probe sets showing inter-platform hit biases were analyzed through ANOVA and Tukey’s HSD test (Stats R package).

### Gene ontology analysis

In order to test whether any of the three copy number detection platforms (aCGH, Illumina-seq, PacBio-seq) were biased toward certain gene types, we ran GO enrichment analysis for subsets of genes identified as gains by all combinations of platforms using the full set of genes detected as gains as background for each respective species. We also tested all candidate gene gains from any platform/species against the all annotated genes as a background set for comparison of our full gene set with other studies. Due to the design of the array, genes were considered to show inter-species copy number differences if one or more of three representative probes for that gene was found as a gain. Therefore, a single gene might fall into different platform-specific sets due to information from different probes. Enrichment analyses were performed in BLAST2GO v5.2.5 [27] and significant results are reported at FDR<0.05.

## Results and discussion

### Probe sequence alignments

A similar number of probe sequences had at least one perfect alignment to the Illumina-based assemblies (*O*. *niloticus* (On): 130,956; *M*. *zebra* (Mz): 100,826) and the PacBio-based genome assemblies (On: 130,679; Mz: 100,192). The greater number for *O*. *niloticus* likely reflects the *O*. *niloticus* species bias in probe design based on quality and coverage differences between assemblies. The intersection of these four sets includes 98,090 probe sequences that had at least one perfect hit in each of the four assemblies, representing 72.9% of the total probe sequences tested. The majority of this intersecting set, 88,871 (90.6%), had only one hit in each of the four genome assemblies, representing putative single copy sequences. However, 9,219 (9.4%) probe sequences had two or more perfect matches in one or more of the assemblies, thus representing possible copy number variation. Only 88 of these probes with multiple alignments in genome assemblies had equal numbers of hits across assemblies. The technology bias towards more alignments in the PacBio-based assemblies (On: 2,282, Mz: 5,536) than in Illumina-based assemblies (On: 928, Mz: 385) (Kruskal-Wallis rank sum test, P < 2.2e-16; Mz: df = 36, *X*^2^ = 341.75; On: df = 37, *X*^2^ = 845.49) reveals loci that were “collapsed” in Illumina assemblies, thus obscuring true inter-species structural variation. Likely due to the lower quality of the original *M*. *zebra* Illumina assembly, more loci appear collapsed in *M*. *zebra* than in *O*. *niloticus*.

Only 928 *O*. *niloticus* and 385 *M*. *zebra* probes had a greater number of perfect alignments in Illumina assemblies than in PacBio assemblies, which may reflect within-species variation given that different individuals were sequenced by each platform. Alternatively, it may also represent missed alignments due to higher error rates in PacBio combined with our strict threshold of requiring perfect assemblies. While *O*. *niloticus* samples for all three platforms derive from the University of Stirling line of clones, individual samples may harbor *de novo* mutations. For *M*. *zebra*, a single wild individual was used for the Illumina assembly, a pool of individuals were used for the PacBio assembly, and three unique individuals were used for aCGH. Thus, individual variation could contribute to the reduced overlap in copy number detection among platforms seen for *M*. *zebra*.

### Inter-species CNV detection methods

**Fig 1:**
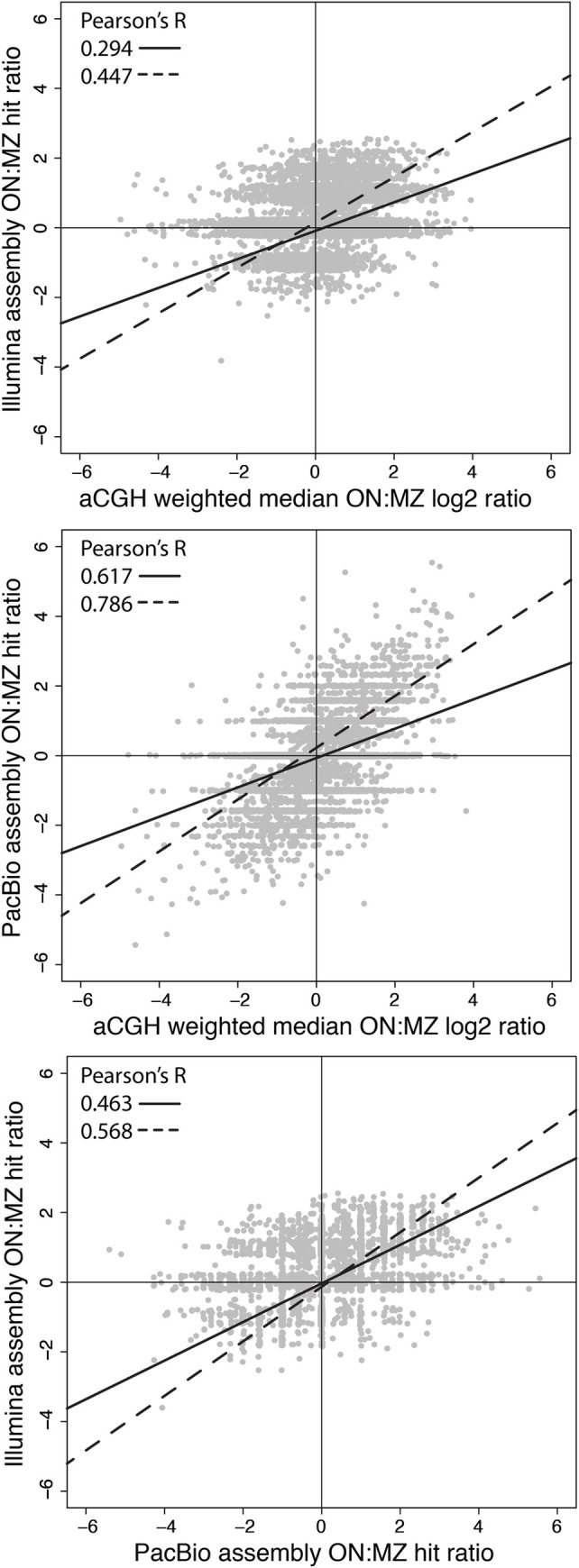
PacBio hit ratios correlate better with aCGH hybridization ratios than Illumina hit ratios. Pearson’s correlations and linear regressions represent all 9,219 probes with 2 or more hits in at least one genome assembly (solid line) and those detected with different copy numbers in the two plotted platforms (dotted line). Points are jittered to better show high density of overlapping points (particularly around 0 ratios) when log_2_ ratios are calculated from discrete integers of BLAST-hit counts.

Focusing on the 9,219 probe sequences representing possible copy number variable loci, we calculated inter-species log_2_ (hybridization or hit) ratios to estimate the relative number of copies of a particular probe sequence in *O*. *niloticus* compared to *M*. *zebra* for each platform (aCGH, Illumina-seq, PacBio-seq). The strongest correlation was between aCGH and PacBio log_2_ ratios (Pearson’s R = 0.617, t = 75.304, df = 9217) while PacBio and Illumina had the second strongest correlation (Pearson’s R = 0.463, t = 50.212, df = 9217) and aCGH and Illumina had the weakest correlation (Pearson’s R = 0.294, t = 29.526, df = 9217) (P < 2.2e-16 for all correlations). These correlations are made more robust by removing neutral and near-neutral probes for the two platforms compared (Fig 1, S1 File). The strong correlation between inter-species aCGH and PacBio log_2_ ratios underscores the fact that PacBio technology validates a large set of copy number variable loci missed by short-read technologies, yet these loci could still be detected by aCGH [5, 28]. Segmentation analysis was not performed here for aCGH results, which allowed for more accurate comparisons across platforms at a probe level. At this fine-scale resolution, we found sets of probes showing aCGH-based inter-species differences overlapping with sets of probes showing both inter-species differences among NGS platforms and inter-platform differences within a species.

**Fig 2:**
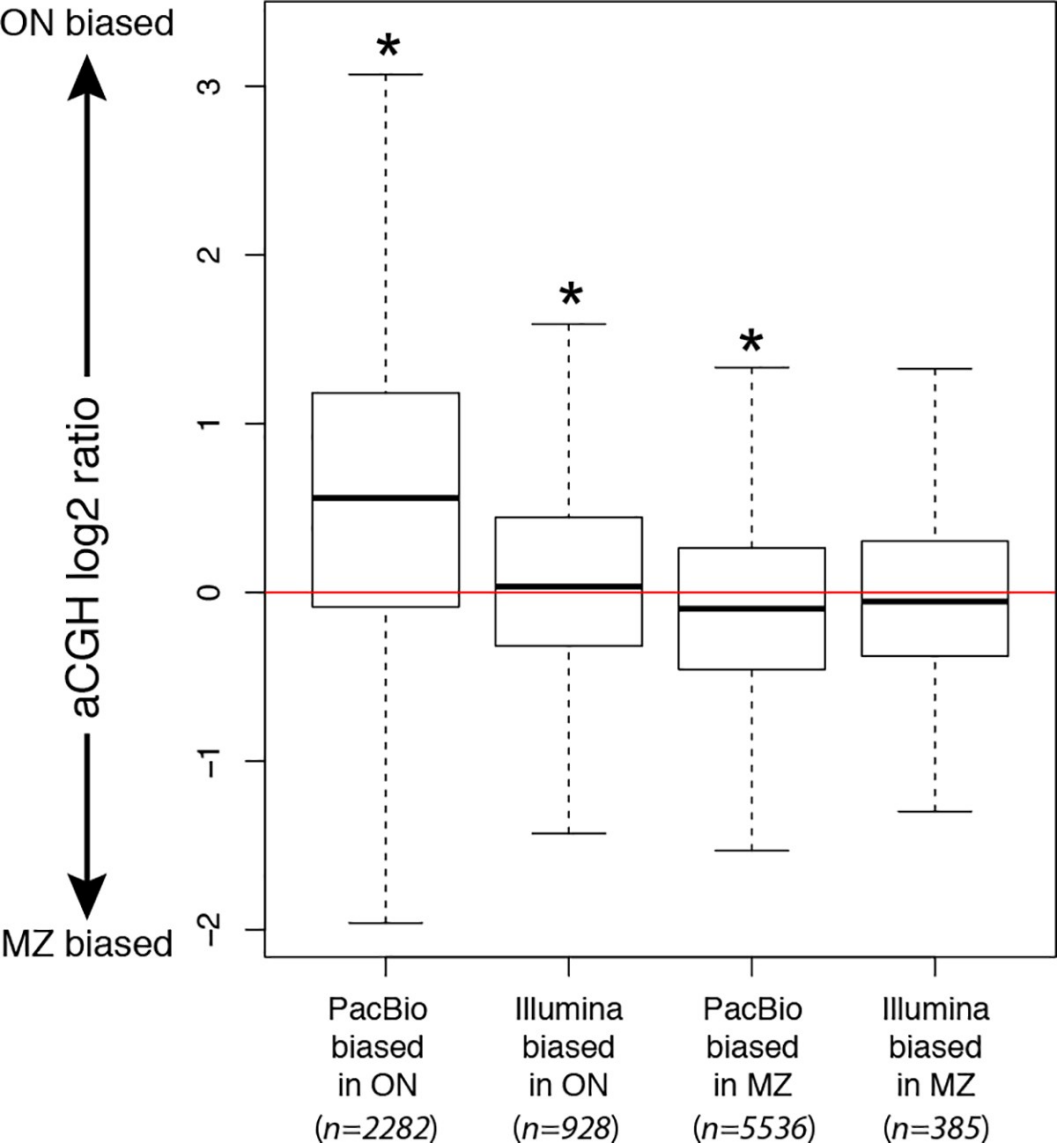
Intra-species NGS platform bias and relationship to inter-species aCGH bias. aCGH log_2_ hybridization ratios of probes with variable numbers of hits between sequencing technologies are significantly different from the full distribution of aCGH ratios in the direction of PacBio biased probes. *Significance is indicated for pairwise Tukey’s HSD tests at FDR<0.05.

We also tested whether those loci that are expanded in the PacBio-based genome assemblies are different between species according to aCGH log_2_ ratios (Fig 2). For *O*. *niloticus*, the PacBio-biased set of probe sequences had a higher average median aCGH log_2_ hybridization ratio than the full set of probe sequences (n = 2,282; Tukey’s HSD: adj. P < 2e-16) and for *M*. *zebra* the PacBio-biased set of probe sequences had a lower average median aCGH log_2_ hybridization ratio than the full set of probe sequences (n = 5,536; Tukey’s HSD: adj. P < 2e-16). Interestingly, the Illumina-biased set of probe sequences also showed the expected species bias in aCGH for *O*. *niloticus* (n = 928; Tukey: adj. P = 1.48e-5) and, though not significantly, for *M*. *zebra* (n = 385; Tukey: adj. P = 0.246).

**Fig 3:**
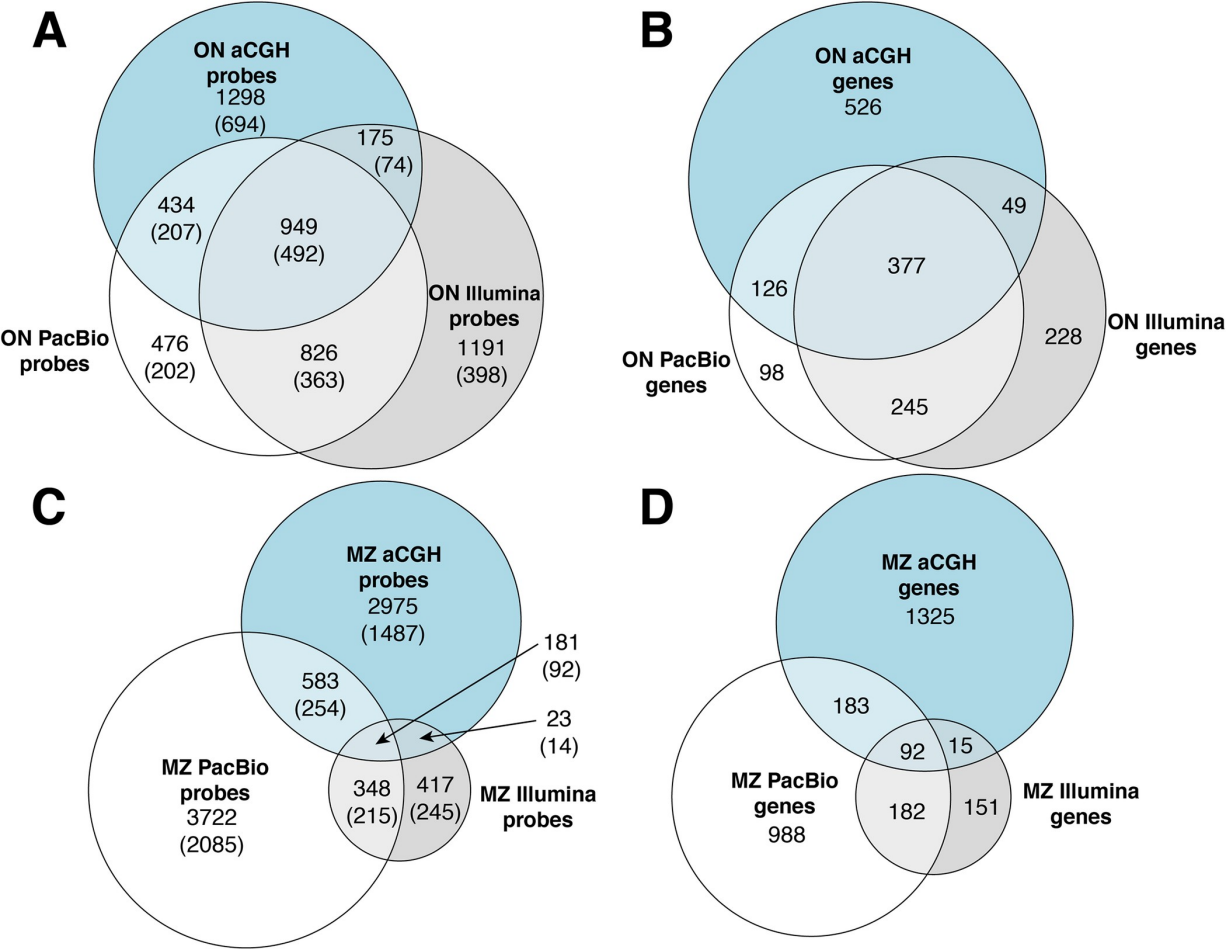
Euler Diagram showing counts of probes and genes detected as copy number variable for each technology. (A) and (B) are counts of probes and the genes they represent, respectively, that appear to have *O*. *niloticus* bias by at least one of the three platforms, while (C) and (D) are counts of probes and genes, respectively, that appear to have *M*. *zebra* bias by at least one platform. Probe counts listed in (A) and (C) include both intergenic and genic probes while counts in parentheses include genic probes only. For a probe to be biased towards a species in NGS assemblies, there must be more exact sequence matches in one assembly than the other. To account for noise in aCGH results, probes must be species biased after filtering of near-neutral log_2_ hybridization ratios (+/-0.8).

While many probe sequences were detected as concordant gains across all three platforms (On: 949, Mz: 181), each technology also identified a unique set (Fig 3) [8]. Within-species variation for *M*. *zebra* may explain a portion of these unique probe sets seen as gains in *M*. *zebra*, or losses that would manifest as relative gains in *O*. *niloticus* despite clonal line samples for the latter species. The aCGH-specific probes likely represent copy number gains with sequence divergence that are not captured by our 100% BLASTn threshold used to calculate hit ratios [5, 28] (S1 Fig). Some of the Illumina or PacBio platform-specific probes may represent higher copy number sequences in which the ratio between species is not dramatic and would be missed by our conservative +/-0.8 log_2_ aCGH threshold. The probe-sets identified by aCGH had greater overlap with PacBio-identified inter-species differences than with those identified by Illumina. Overall, probes representing copy number gains in *O*. *niloticus* showed more reciprocal overlap between platforms than those in *M*. *zebra* likely due the lower quality of the *M*. *zebra* Illumina assembly, also contributing to underrepresentation of *M*. *zebra* sequences in array design.

The patterns of overlap for the number of gene gains was similar to, but lower than, those for the number of probe sequences both because probe sequences can be intergenic and genes are represented by multiple probes (S1 Table). This genic content further reinforces the notion that many of the multicopy sequences collapsed by short-read sequencing/assembly may be important in understanding species divergence as genomic structural variation within the species provides the substrate for evolution. aCGH can be used to detect both the intra-species and inter-species variation upon which selection can act leading to divergence and diversification [29–34]. In several instances these genomic regions have been shown to underlie adaptive phenotypes [35, 36] and reflect phylogenetic relationships [37].

### Sequence characteristics of platform biases

To examine whether nucleotide composition played a role in which probe sequences were detected as gains by each platform, we assessed single nucleotide composition, dinucleotide composition, and overall G/C or A/T content. We found that aCGH-, Illumina-, and PacBio-exclusive probe sequence sets were all significantly different from each other in nucleotide composition (pairwise permutation MANOVA FDR = 0.0099). PCA was run to determine which nucleotide composition statistics appear to explain the most variation across groups (S2 Table, S2 Fig), and PC1 was heavily weighted by G/C vs. A/T content and explains 24.5% of the variance in the dataset with PacBio probe hits having a slight bias towards a higher GC content. PC2 was heavily weighted by variation in A nucleotide frequency along with related A-containing dinucleotides, explaining 18.8% of variation, and PC3 was heavily weighted by variation in C/A vs. G/T nucleotide frequency, explaining 16.6% of the variance. No other components explained more than 6% of the variance and appear relatively unimportant in identifying platform biases based on nucleotide composition.

Post-hoc univariate ANOVAs were performed for each sequence characteristic in a pairwise comparison of platform-specific species-biased probes. These results confirmed the nucleotide/dinucleotide characteristics seen in PCAs (S2 Table). After performing Bonferroni correction, 2 sequence characteristics differentiate aCGH from Illumina, 14 sequence characteristics differentiate aCGH from PacBio, and 6 differentiate Illumina from PacBio. The most prominent pattern shows PacBio is significantly differentiated from both aCGH and Illumina probes by G/C and inversely A/T content, which are the terms responsible for variation along PC1. PacBio detected probes are most differentiated from aCGH detected probes by A content, but this signal is less significant in the comparison of PacBio vs. Illumina making G/C variation the strongest pattern overall. This pattern in aCGH probes reflects PCA loadings, which indicate that A content is responsible for the most variation along PC2. aCGH and Illumina probes are differentiated by the fewest nucleotide/dinucleotide identities, but vary most in their GC dinucleotide content. GC content is more specific in its nucleotide arrangement and is separate from overall G/C content found to differentiate PacBio from both other platforms. There have been a number of studies showing that G/C content can influence hybridization efficiency of arrays or library preparation and assembly steps for NGS sequencing technologies [38, 39]. The variation found in A content is interesting in that it parallels results from previous studies showing deficits in adenine content in next-gen sequencing libraries built with both blunt-end or AT overhang ligation [40]. Given the array probes compared here were built using the Illumina assemblies as templates, these results suggest aCGH technologies may be better suited to detect real copy number variation in A-rich regions that are underrepresented in short read sequencing libraries.

### Function of copy number variable genes

To determine whether the different platforms might lead to different functional inference (e.g. be biased toward detection of specific gene families) we ran Gene Ontology enrichment analyses on the sets of copy number variable genes identified by all combinations of platforms in either species. Enrichment results for the full set of gene gains from any platform in either species (using all annotated genes as background) closely parallel earlier aCGH studies [8, 41–46] in identifying GO terms associated with adaptation to diverse environments and categories of genes known to proliferate. These terms include G-protein coupled receptor pathways, detection of chemical stimulus, olfactory receptor activity, monooxygenase activity, olfactory receptor activity, integral component of membrane, iron ion binding, and oxidoreductase activity (Table 1), with the first four GO terms also detected in our previous broader aCGH study [8]. Enrichment analyses performed on full sets of genes detected as gains by each technology for each species (using all species-specific candidate gene gains as background) yielded no significantly enriched GO terms, revealing no obvious functional biases. For genes detected exclusively by a single technology (*i*.*e*. no overlap with other platforms), aCGH showed only modest enrichment (FDR < 0.05) for only four overlapping terms: regulation of transcription and nucleus in both species, and sequence-specific DNA binding in *O*. *niloticus* and intracellular signal transduction in *M*. *zebra*, with only one significant at FDR < 0.01 (regulation of transcription in *O*. *niloticus*). This result could either reflect sequence hybridization bias on the array or the gene-centric bias of the array design, but lack of enrichment results for full gene sets from each platform suggests that the platforms largely coalesce around the detection of similar sets of genes.

**Table 1:** Gene Ontology (GO) enrichment for gene subsets.

GO ID	GO Name	GO Cat	FDR	% Test Set	% BG Set
***All candidate copy number variable genes vs*. *full genome annotation***					
GO:0004930	G protein-coupled receptor activity	MF	3.31E-11	6.8%	3.0%
GO:0007186	G protein-coupled receptor signaling pathway	BP	8.52E-09	7.1%	3.6%
GO:0050911	detection of chemical stimulus involved in sensory perception of smell	BP	1.77E-03	0.9%	0.2%
GO:0004497	monooxygenase activity	MF	1.77E-03	1.2%	0.4%
GO:0004984	olfactory receptor activity	MF	1.77E-03	0.9%	0.2%
GO:0016021	integral component of membrane	CC	2.56E-03	30.6%	25.8%
GO:0005506	iron ion binding	MF	4.48E-03	1.6%	0.6%
GO:0016705	oxidoreductase activity, acting on paired donors, with incorporation or reduction of molecular oxygen	MF	4.77E-02	1.3%	0.5%
***aCGH-only O*. *niloticus gene gains vs*. *all predicted O*. *niloticus gene gains***					
GO:0006355	regulation of transcription, DNA-templated	BP	9.74E-03	4.2%	0.4%
GO:0043565	sequence-specific DNA binding	MF	1.56E-02	2.8%	0.0%
GO:0005634	nucleus	CC	4.00E-02	6.7%	1.9%
***aCGH-only M*. *zebra gene gains vs*. *all predicted M*. *zebra gene gains***					
GO:0035556	intracellular signal transduction	BP	3.91E-02	5.1%	1.5%
GO:0005634	nucleus	CC	4.48E-02	7.9%	3.4%
GO:0006355	regulation of transcription, DNA-templated	BP	4.57E-02	6.1%	2.2%

GO category abbreviations are MF for Molecular Function, BP for Biological Process, and CC for Cellular Component. Percentage columns report the percentage of the genes for each GO term within test and background (BG) gene sets.

## Conclusion

The primary goal of our study was to demonstrate the extent to which an array-based platform (aCGH) can identify genomic loci that are collapsed in short-read genome assemblies by taking advantage of two cichlid species for which both Illumina and PacBio genome assemblies are available for comparison to inter-species aCGH data. Our study shows that relative sequence copy numbers from PacBio genome assemblies correlate better with aCGH results than either technology does with estimates from the Illumina-based assemblies. Due to the design of our microarray, our results are biased toward loci that are present in ten or fewer copies in Illumina assemblies rather than highly repetitive elements. The larger number of sequence gains detected in *M*. *zebra* compared to *O*. *niloticus* is also likely impacted by the array probe design based on the different levels of completeness of the Illumina cichlid genomes. We assessed platform biases further and found them to be minimal at a functional and sequence characteristic level, although we identify nucleotide characteristics that potentially underlie such biases. Overall, these results demonstrate that aCGH remains a valid and effective approach for between-species [30, 31] or within-species [29] CNV studies that could be applied for population level studies [47, 48]. A complete understanding of the molecular basis for adaptive natural selection, speciation, and population level structural variation greatly benefits from detection of copy number differences within and between species utilizing the multiple platforms explored here.

## Supporting information

S1 Fig Impact of BLASTn percent identity threshold on detection of species-biased probes between platforms. Figures show reciprocal proportional overlap of probes detected as gains between aCGH and sequencing platforms for (A) *M*. *zebra* and (B) *O*. *niloticus*. The x-axis represents BLASTn hit % identity cutoffs used to generate probe sequence counts for both sequencing platforms. All probes were required to have at least one 100% identity hit in each of the four genome assemblies considered. For *M*. *zebra*, the reciprocal overlaps do not appreciably change as the threshold is lowered. For *O*. *nilocitus*, the overlaps of aCGH with sequencing platforms increase as we lower the BLASTn threshold while the overlaps of sequencing platforms with aCGH decrease. This pattern is due to the expansion of the Illumina and PacBio sets adding more non-concordant than concordant hits. There is a slight increase in both reciprocal overlaps with a slight reduction in threshold (98%), however these results show that choosing 100% as a conservative threshold to generate BLASTn hit counts provided sufficient concordance between datasets while still allowing for accurate downstream assessment of platform biases based on known nucleotide composition of probes.
(TIF)

S2 Fig Nucleotide composition PCA for platform-exclusive probes. Principal component analysis (PCA) plots representing distributions of probes detected as gains in either *M*. *zebra* or *O*. *niloticus* by only one of three platforms. Panel (A) shows PCs 1 and 2 and panel (B) shows PCs 2 and 3. Contribution of all nucleotide composition statistics are superimposed on each plot to visualize potential platform biases. While all platform-exclusive probe sets largely overlap, probes with higher G/C content appear more likely to be detected as gains in PacBio assemblies than by Illumina or aCGH along PC1. Additionally, aCGH-exclusive probe sets have slightly higher A-content than either sequencing platform exclusive set as represented by PC2 and PC3.
(TIF)

S1 File Linear regression output. (TXT)

S1 Table Gene probe information. (XLSX)

S2 Table Nucleotide composition PCA output & pairwise MANOVA results. (XLSX)
