# Supplementary figures and images for: Correspondence of aCGH and long-read genome assembly for detection of copy number differences: A proof-of-concept with cichlid genomes
Source: PLoS One. 2021 Oct 7;16(10):e0258193. doi: 10.1371/journal.pone.0258193 (PMC8496808; doi:10.1371/journal.pone.0258193)

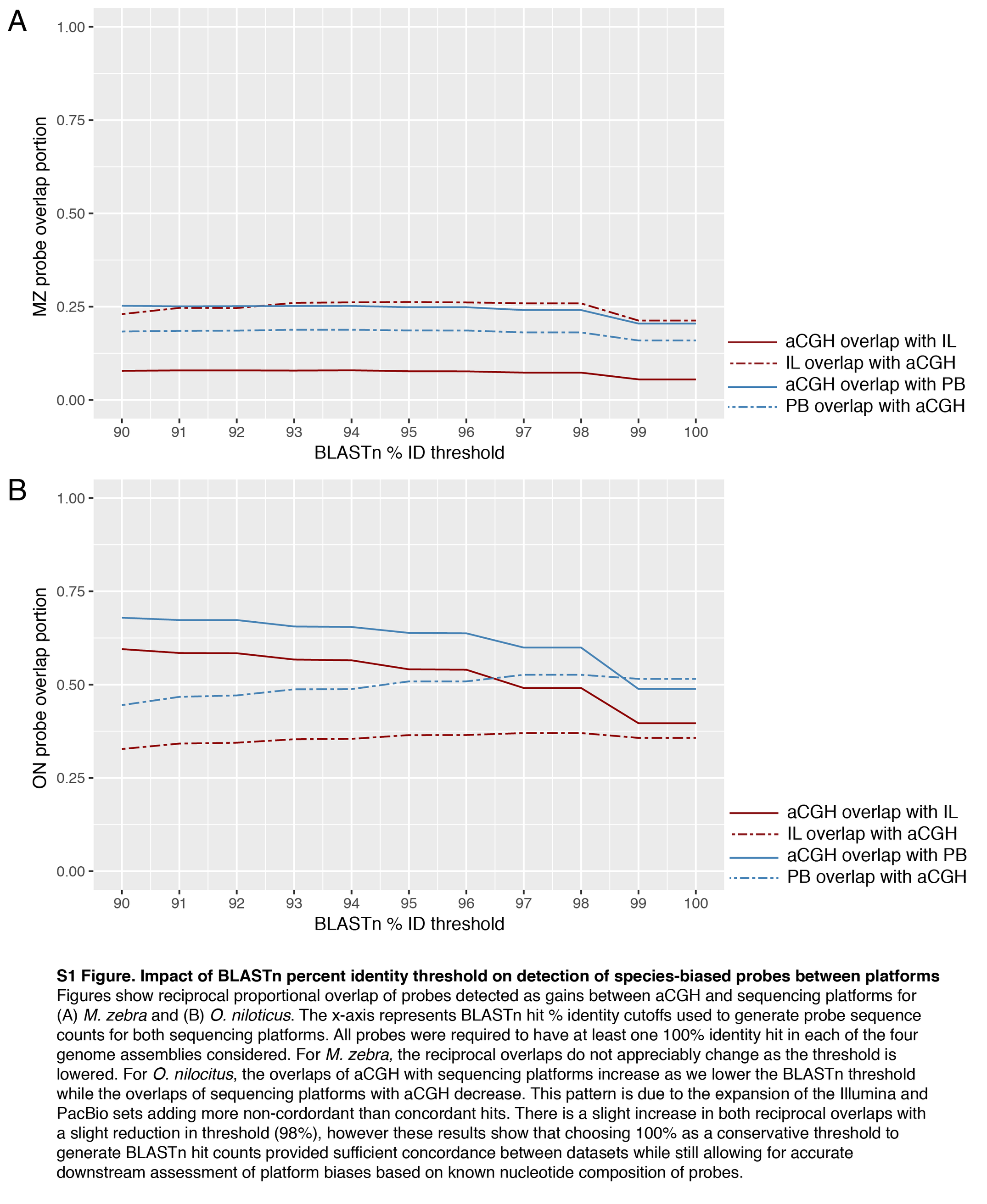

Supplement: S1 Fig — Figures show reciprocal proportional overlap of probes detected as gains between aCGH and sequencing platforms for (A) M. zebra and (B) O. niloticus. The x-axis represents BLASTn hit % identity cutoffs used to generate probe sequence counts for both sequencing platforms. All probes were required to have at least one 100% identity hit in each of the four genome assemblies considered. For M. zebra, the reciprocal overlaps do not appreciably change as the threshold is lowered. For O. nilocitus, the overlaps of aCGH with sequencing platforms increase as we lower the BLASTn threshold while the overlaps of sequencing platforms with aCGH decrease. This pattern is due to the expansion of the Illumina and PacBio sets adding more non-concordant than concordant hits. There is a slight increase in both reciprocal overlaps with a slight reduction in threshold (98%), however these results show that choosing 100% as a conservative threshold to generate BLASTn hit counts provided sufficient concordance between datasets while still allowing for accurate downstream assessment of platform biases based on known nucleotide composition of probes. (TIF) [file pone.0258193.s001.tif]

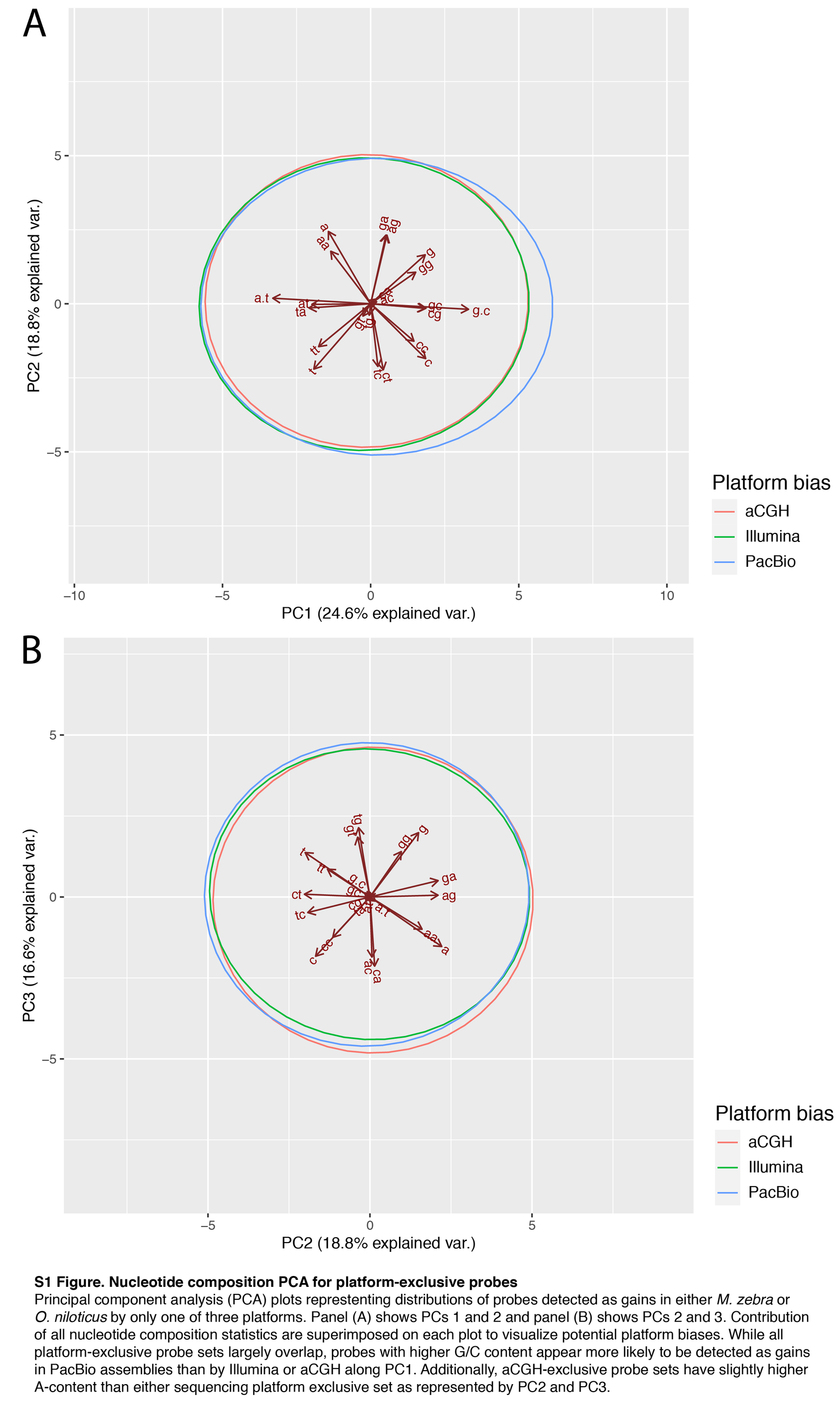

Supplement: S2 Fig — Principal component analysis (PCA) plots representing distributions of probes detected as gains in either M. zebra or O. niloticus by only one of three platforms. Panel (A) shows PCs 1 and 2 and panel (B) shows PCs 2 and 3. Contribution of all nucleotide composition statistics are superimposed on each plot to visualize potential platform biases. While all platform-exclusive probe sets largely overlap, probes with higher G/C content appear more likely to be detected as gains in PacBio assemblies than by Illumina or aCGH along PC1. Additionally, aCGH-exclusive probe sets have slightly higher A-content than either sequencing platform exclusive set as represented by PC2 and PC3. (TIF) [file pone.0258193.s002.tif]
